# Characterization of *Salmonella* endolysin XFII produced by recombinant *Escherichia coli* and its application combined with chitosan in lysing Gram-negative bacteria

**DOI:** 10.1186/s12934-022-01894-2

**Published:** 2022-08-23

**Authors:** Shuhang Zhang, Yan Chang, Qing Zhang, Yingbo Yuan, Qingsheng Qi, Xuemei Lu

**Affiliations:** 1 State Key Laboratory of Microbial Technology, Shandong University, Qingdao, 266237 China; 2 Institute of Animal Science and Veterinary Medicine, Shandong Academy of Agricultural Sciences, Jinan, 250100 China

**Keywords:** *Salmonella*, Endolysin, Heterologous expression, Gram-negative pathogen, Chitosan, Bactericidal activity

## Abstract

**Background:**

*Salmonella* is a common foodborne pathogen, which can cause intestinal diseases. In the last decades, the overuse of antibiotics has led to a pandemic of drug-resistant bacterial infections. To tackle the burden of antimicrobial resistant pathogens, it is necessary to develop new antimicrobial drugs with novel modes of action. However, the research and development of antibiotics has encountered bottlenecks, scientific hurdles in the development process, as well as safety and cost challenges. Phages and phage endolysins are promising antibacterial agents that can be used as an alternative to antibiotics. In this context, the expression of endolysin derived from different phages through microbial cells as a chassis seems to be an attractive strategy.

**Results:**

In this study, a new endolysin from the *Salmonella* phage XFII-1, named XFII, was screened and obtained. The endolysin yield exceeded 100 mg/mL by heterologous expression from *E. coli* BL21 and short induction. The endolysin XFII exhibited high bactericidal activity at a concentration of 0.5 μg/mL and reduced the OD_600_ nm of EDTA-pretreated *E. coli* JM109 from 0.8 to 0.2 within 5 min. XFII exhibited good thermo-resistance, as it was very stable at different temperatures from 20 to 80℃. Its bactericidal activity could keep constant at 4 °C for 175 days. In addition, the endolysin was able to exert lytic activity in eutrophic conditions, including LB medium and rabbit serum, and the lytic activity was even increased by 13.8% in 10% serum matrices. XFII also showed bactericidal activity against many Gram-negative bacteria, including *Salmonella*, *E. coli*, *Acinetobacter baumannii,* and *Klebsiella pneumoniae*. Surprisingly, the combination of endolysin XFII and chitosan showed a strong synergy in lysing *E. coli* and *Salmonella* without EDTA-pretreatment, and the OD_600_ nm of *E. coli* decreased from 0.88 to 0.58 within 10 min.

**Conclusions:**

The novel globular endolysin XFII was screened and successfully expressed in *E. coli* BL21. Endolysin XFII exhibits a broad lysis spectrum, a rapid and strong bactericidal activity, good stability at high temperatures and under eutrophic conditions. Combined with chitosan, XFII could spontaneously lyse Gram-negative bacteria without pretreatment. This work presented the first characterization of combining endolysin and chitosan in spontaneously lysing Gram-negative bacteria in vitro.

**Supplementary Information:**

The online version contains supplementary material available at 10.1186/s12934-022-01894-2.

## Introduction

*Salmonella* is a common Gram-negative food-borne pathogen that can cause acute gastroenteritis or virulent infection in humans and food-producing animals, characterized by diarrhea, abdominal pain, nausea, vomiting, fever and other diseases [1]. *Salmonella* is carried and excreted by animals and spreads to the environment, feeds, water, and food, and then to humans through poultry, meat, milk, and eggs [2–4]. Among them, *Salmonella enteritidis* is one of the most common causes of food-borne illness in North America. People with no immune response and infants are susceptible to severe enteritis, leading to systemic infection and death [5, 6]. It is a kind of pathogenic bacteria threatening human health. However, the overuse of antibiotics has led to an increase in drug-resistant bacterial infections, so it is urgent to find new antibacterial drugs [7]. Bacteriophage-derived proteins such as endolysins provide one potential solution to this pressing issue. Unlike traditional broad-spectrum antibiotics, endolysins have a number of advantages, including high efficiency, no disruption of non-target cells and animal cells, enzyme proteins are easy to produce and modify, and antibodies do not affect their antimicrobial activity and effectiveness against antibiotic-resistant bacteria [8, 9]. In addition, the target specificity of endotoxin makes its antibacterial spectrum wider than that of phage. These characteristics, in particular, make them promising alternatives to antibiotics [9, 10]. In this context, the expression of endolysin from different phages via microbial cells as a chassis has attracted the attention of an increasing number of researchers [11–14]. A number of *Salmonella*-specific endolysins have been identified and characterized recently (Additional file 1: Table S1). However, a major problem is the low expression of most of the reported endolysins, a point that could dissuade the industrial production of these enzymes [15, 16]. In addition, the majority of endolysins exhibit activity involving the aid of outer membrane permeabilizers. For example, LysWL60 with 5% (v/v) chloroform and SPN9CC with 100 mM EDTA [17, 18]. However, some endolysins, such as LysSTG2, have synergistic effects with slightly acidic hypochlorous in controlling *Salmonella typhimurium*, suggesting that cytotoxic outer membrane permeabilizers would be avoided in some cases [19]. While satisfactory progress has been made in the study of Gram-positive bacteria endolysins, the study of endolysins for Gram-negative bacteria faces a major challenge. The outer membrane prevents exogenous endolysins from contact with the peptidoglycan. However, after continuous attempts and efforts by researchers [20–22]. The challenge of Gram-negative endolysins has been solved by: (1) identifying natural endolysins capable of penetrating the outer membrane [23], (2) synergizing endolysins with outer membrane penetrators such as EDTA, chloroform, cationic peptides, and bacteriostatic agents [20, 24–27], or (3) fusing endolysins with a segment of peptide [28, 29]. As a potential antimicrobial agent, chitosan exerts a strong bactericidal effect on foodborne pathogens, and has been widely used as a food preservative [30, 31]. However, the combined effects of chitosan and endolysins on Gram-negative bacteria have not yet been reported.

The stability of the endolysins in the practical application environment has an important influence on their bactericidal effect. On one hand, the inherent thermal stability of protein is a decisive characteristic for its practical application [32]. As temperature-tolerant proteins are easier to handle and retain their activity for longer periods of time [33]. However, the stability of free enzymes is poor. Further comparison shows that the resistant temperature of most of them was below 55 °C as shown in Additional file 1: Table S1. Only endolysin LysSE24 and LysWL59, retain partial activity at 80 °C [17, 34]. Therefore, the development of some temperature resistant endolysin preparations can fundamentally solve the problem of poor thermal stability. On the other hand, considering that the clinical application of endolysins is not only environmental disinfection, but also food disinfection and in vivo sterilization, it is important to have stable bactericidal activity under eutrophic conditions. However, reports showed that the killing activity of most endolysins is significantly reduced or lost under eutrophic conditions such as LB medium and serum [35, 36]. To our knowledge, anti-staphylococcal Lysin CF301 is the only lysin that exhibits substantially greater potency in human blood [37, 38]. Till now, there has been no report on the bactericidal activity of the *Salmonella* endolysin under eutrophic conditions.

In this study, the gene encoding XFII, an endolysin from *Salmonella*-lytic bacteriophage XFII-1, was cloned, over-expressed, and characterized. Our results verified the stability of the endolysin including high yield, high activity, thermo-resistance, storage periods, and stability under eutrophic conditions. In addition, the antimicrobial activity of XFII in synergy with chitosan against in *E. coli* and *Salmonella *in vitro was analyzed. Information obtained from studying the endolysin XFII will be useful in the development of promising phage-based biocontrol agents with bactericidal activity against foodborne pathogens.

## Results

### Cloning, expression, and purification of endolysin derived from bacteriophage XFII-1

**Fig. 1:**
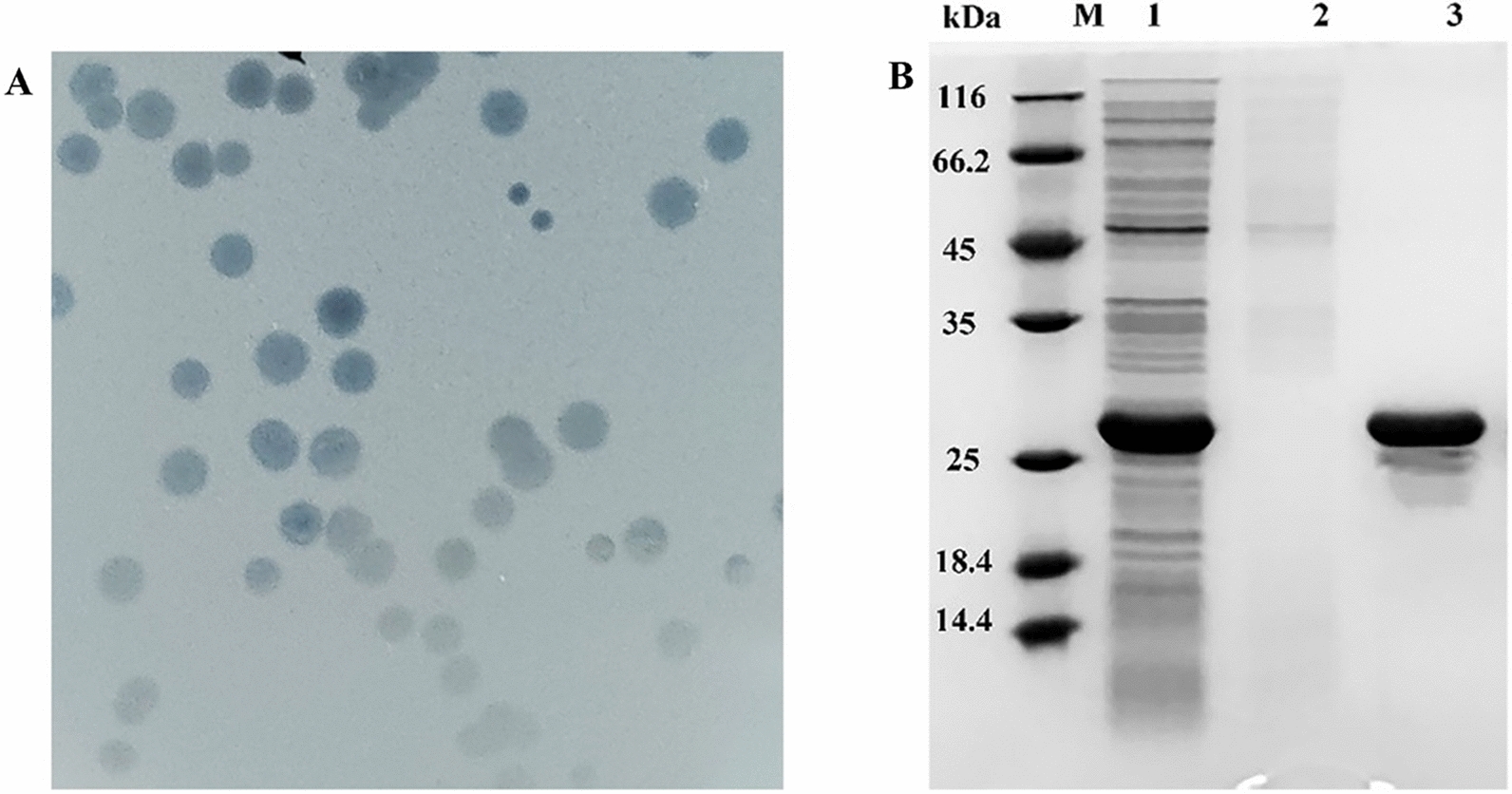
Expression of endolysin XFII. **A** Bacteriophage plaques of XFII diluted several times. **B** SDS-PAGE profiles of extracts of *E. coli* BL21 expressing endolysin XFII. Lanes: M, protein ladder; lane 1, the supernatant of bacterial lysate of the native protein; lane 2, the supernatant of the culture medium of the native protein; lane 3, the XFII endolysin (25.4 kDa) was purified by Ni–NTA affinity chromatography

On soft agar plates with the host *Salmonella* growth, phages XFII-1 can lyse or restrict the growth of infected bacteria, forming intense bacteriophage plaques (Fig. 1A)*.* Subsequently, the whole genome of *Salmonella* phage XFII-1 was sequenced and annotated. The genome sequence of phage XFII-1 contained 58 open reading frames, of which ORF21 was identified as a putative endolysin gene encoding a protein (XFII) consisting of 162 amino acids (accession no.: MZ983433). The endolysin coding region was cloned into expression vector pET-29b( +) to express the recombinant endolysin XFII with a histidine tag at the C-terminus. *E. coli* BL21 containing the recombinant plasmid was induced by IPTG (1 mM) at 16 °C for 4 h. Endolysin XFII was purified by Ni–NTA affinity chromatography, and the recombinant protein was detected by SDS-PAGE (Fig. 1B), which was similar to the predicted band size of about 25.4 kDa, through ExPASy. Most of the endolysin was present in the supernatant of the fragmentation solution, with a single target band and protein yields in excess of 100 mg/L.

### Bioinformatics analysis of XFII

**Fig. 2:**
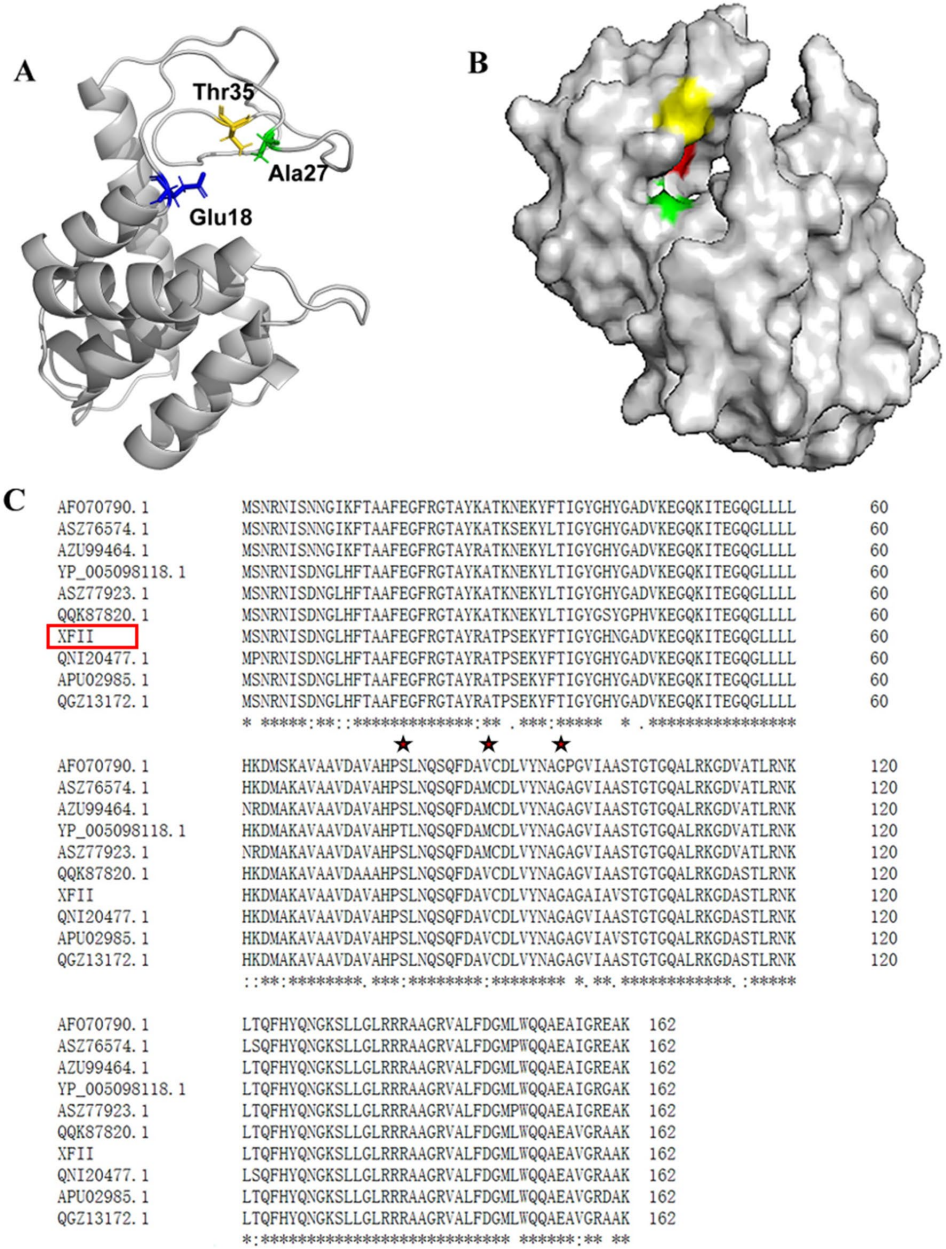
Structural prediction of XFII.. The overall structure of the catalytic module of XFII is exhibited in **A** (drawing) and **B** (Surface). The three catalytic residues are shown and highlighted. **C** Multiple amino acid sequence alignments of XFII with other characterized *Salmonella* endolysins from NCBI GenBank or PDB accession numbers were as follows: *Salmonella* phage LysSE24 (APU02985.1, similarity 98%), *Salmonella* phage PIZ SAE-01E2-12 (QGZ13172.1, similarity 98%), *Salmonella* phage SE2 (YP_005098118.1, similarity 92%,), *Salmonella* phage ST3(ASZ77923.1, similarity 91%), *Salmonella* phage SG2 (ASZ76574.1, similarity 90%), *Salmonella* phage SHWT1 (QNI20477.1, similarity 97%), *Salmonella* phage Lys SP1(QQK87820.1, similarity 93%), *Salmonella* phage ST3 (ASZ77923.1, similarity 91%), *Salmonella* phage ST4 (AFO70790.1, similarity 91%), and *Salmonella* phage TS3 (AZU99464.1, similarity 90%). Conserved amino acids were denoted by stars

The primary and tertiary structure of XFII was bioinformatically analyzed and its possible catalytic residues were identified. XFII was predicted to consist of a single conserved structural domain and was subsequently identified using the NCBI CDD, as belonging to the Lyz endolysin autolysin structural domain family (Accession cd00737), between 10–147 aa, and the conserved domain in query coverage 6–151 aa belongs to the GH24 family of glycoside hydrolases (Accession COG3772). BlastP analysis showed high similarity of *Salmonella* phage endolysins, while XFII was most similar to the recently reported LysSE24 (98% similarity, E-value 6e^−112^) (Fig. 2C). Finally, the tertiary structure of endolysin XFII was simulated according to Alphafold2, XFII was predicted to be a globular protein (Fig. 2B).

### Bactericidal activity of endolysin XFII

**Fig. 3:**
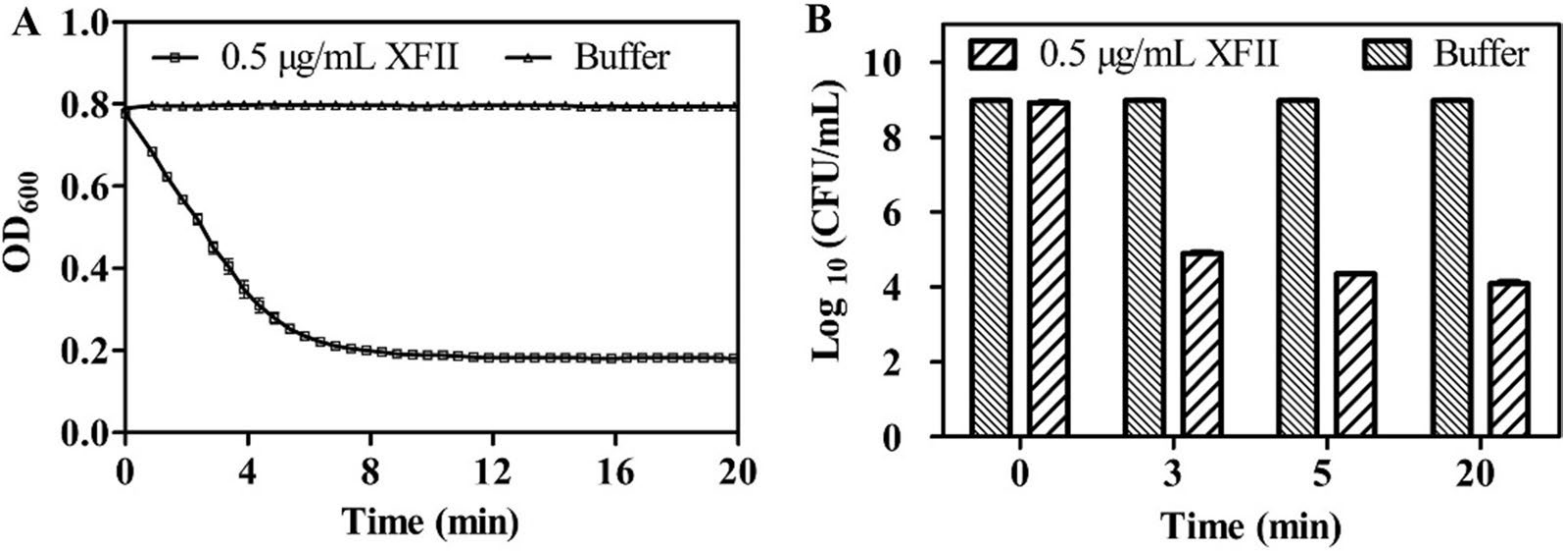
Enzymatic lytic activity of endolysin XFII. **A** Lytic activity detection of endolysin XFII by the turbidity method. **B** Time-killing activity of endolysin XFII against log arithmic phase of *E. coli* JM109 in Tris buffer using measurement by counting colony forming units. Endolysin was added to the substrate at time zero

To determine the lytic activity of endolysin XFII, the turbidity method was used with a slight modification [39]. The addition of purified XFII endolysin alone to *E. coli* cell was impracticable (data not shown) due to its inability to cross the outer membrane of Gram-negative bacteria [20]. However, EDTA-pretreated *E. coli* suspension was found to become rapidly clarified in the presence of 0.5 μg/mL endolysin XFII, reducing the OD_600_ nm from 0.8 to 0.2 within 5 min (Fig. 3A).

Time kill tests showed that endolysin XFII (0.5 μg/mL) killed *E. coli* JM109 by 4.5 log during the first 3 min of incubation and by up to 5.3 log after 20 min of incubation. These results indicate that XFII has strong and rapid bactericidal activity (Fig. 3B).

### Transmission electron microscope (TEM) observation of cell morphology of XFII-treated *E. coli*

**Fig. 4:**
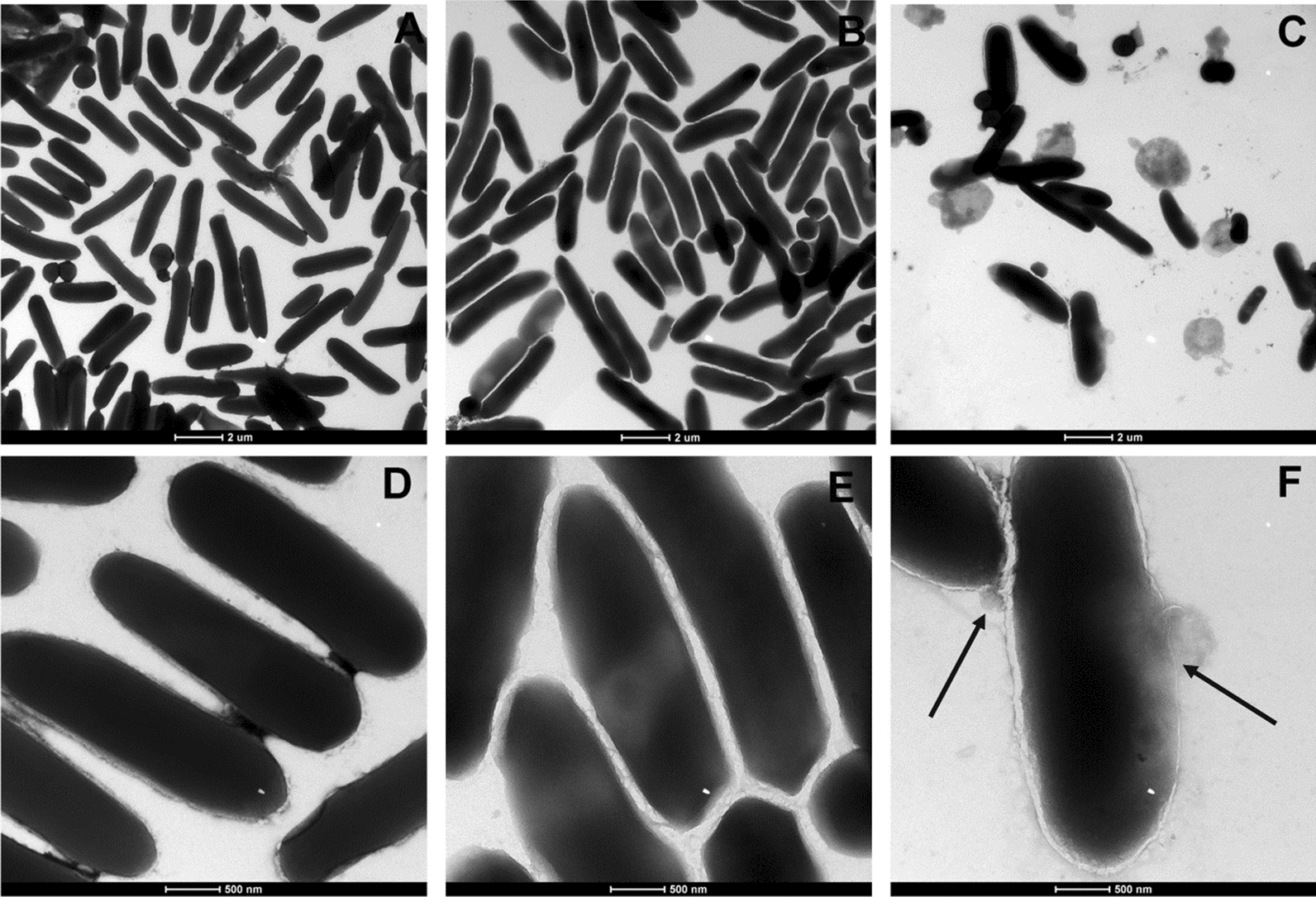
Effect of XFII on the *E.coli* JM109. Transmission electron micrograph (TEM) of *E.coli* JM109 treated with PBS (**A**: 2 µm, **D**: 500 nm), EDTA (**B**: 2 µm, **E**: 500 nm) and 0.5 μg/mL endolysin XFII (**C**: 2 µm, **F**: 500 nm)

In order to study the antibacterial activity of endolysin XFII more intuitively, the morphological changes of *E. coli* were observed by TEM (Fig. 4). In the absence of any treatment, the bacterial cell surfaces are smooth and brilliant (Fig. 4A and D). 100 mM EDTA pretreated *E. coli* had a rod-like morphology with no surface rupture and only the outer membrane became blurred where it may have been distraced (Fig. 4B and E). Compared with the control, after 3 min of treatment with endolysin at 0.5 μg/mL, the cell morphology was distorted, and exhibited bacterial cell wall damage Fig. 4C and F. The number of *E. coli* was significantly lower than that without any treatment and a large amount of cell contents was observed in the microscopic field. These data demonstrated that XFII in excess of 0.5 μg/mL can effectively lyse *E. coli.*

### Optimum pH and temperature conditions for activity of XFII

**Fig. 5:**
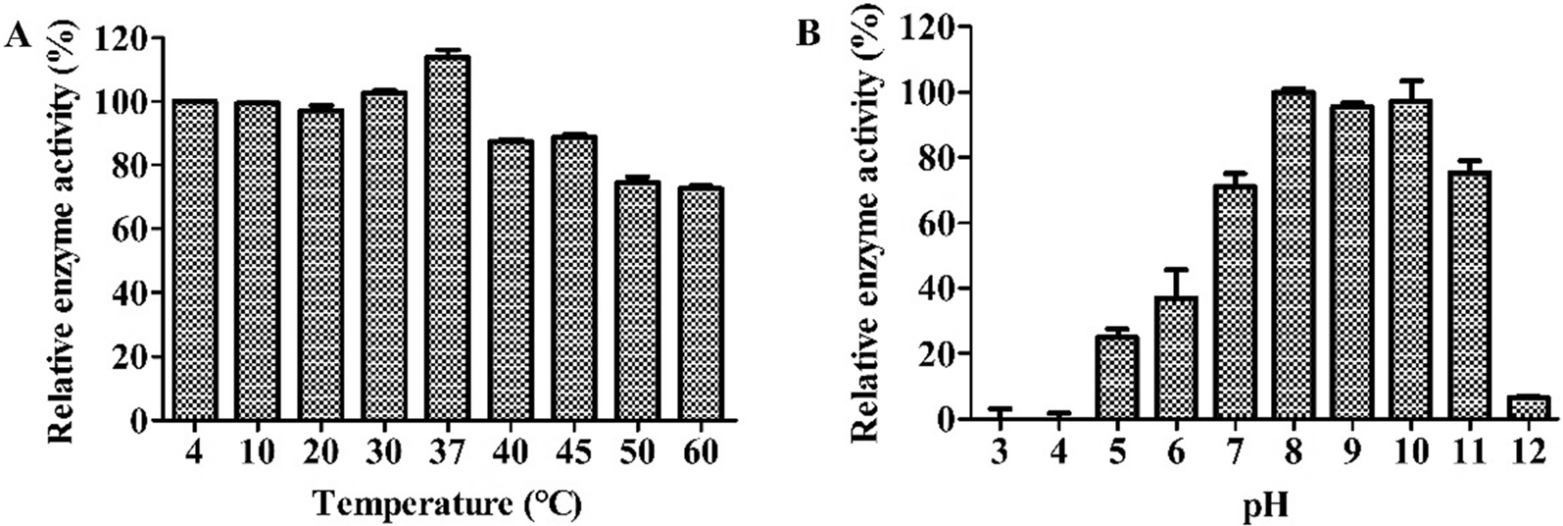
Optimum pH and temperature for the activity of the XFII endolysin. The activity assay was performed at different temperatures (**A**), and in buffers with different pH values (3.0–12.0) (**B**)

The effect of the reaction temperature on the enzymatic activity of XFII was determined. As shown in Fig. 5A, XFII endolysin was active over a range of temperatures (4–60 °C), with the highest activity at 37 °C. The enzyme activity under high temperature reaction conditions at 60 °C decreased to 72.6%. It showed bactericidal activities at neutral and alkaline conditions, with high bactericidal activity at pH 7.0 to 11.0, with the highest activity observed at pH 8.0 (Fig. 5B). The relative bactericidal activity was inhibited at pH 3.0–6.0, with approximately 25% activity retained at pH 5.0. Therefore, it is assumed that XFII is an alkalophilic lyase and that it can be active over a wide range of pH.

### Thermal and storage stability testing of endolysin XFII

**Fig. 6:**
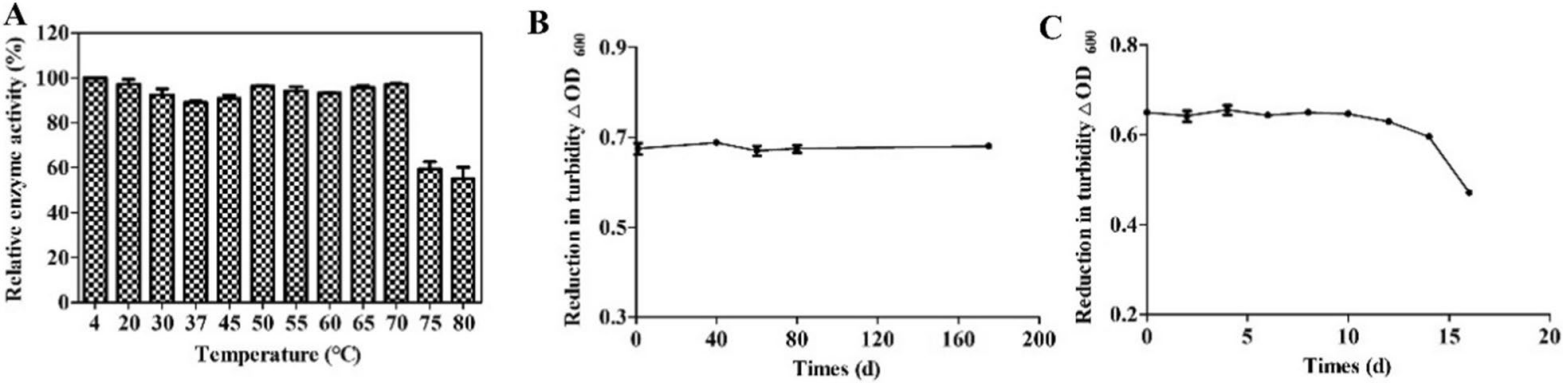
Enzymatic stability of endolysin XFII. **A** The temperature resistance of endolysin XFII was measured by placing it at different temperatures for 2 h. Enzyme activity assay of purified endolysin at 4 °C (**B**) and at room temperature (**C**)

To test the thermal stability of the endolysin, the relative lytic activity of XFII was studied at various temperatures (4–80 °C) for 2 h. Thermal treatment at temperatures below to 70 °C for 2 h did not significantly affect endolysin activity. Fortunately, the bactericidal activity of 80 °C for 2 h was still 54.8% (Fig. 6A).

The purified endolysin XFII was stored at 4 °C in the refrigerator and is shown in Fig. 6B. The bactericidal activity of endolysin XFII kept constant for 175 days at 4 °C, retaining 98.8%, while data for longer storage periods are not tested. When endolysin XFII was stored in room conditions (between 20 and 28 °C), the bactericidal activity was not affected within 14 days. Even after 16 days of storage, the lytic activity was still approximately 72% of its original level (Fig. 6C).

### The lytic spectrum of endolysin XFII

*E. coli* DH5α, *E. coli* JM109, *E. coli* BL21 (DE3), *E. coli* Min O157, *Klebsiella pneumoniae*, *Staphylococcus aureus*, 12 strains of pathogenic *E. coli* and 6 strains of *Salmonella* isolated from the environment were analyzed for susceptibility to the bactericidal spectrum of endolysin XFII (Table 1). Endolysin XFII showed lytic activity against most of the Gram-negative bacteria that were pretreated with 100 mM EDTA, Among these bacteria, the highest activities were observed against *E. coli* strains JM109, DH5α, 19023 and 19019 (from the environment). Several pathogenic *E. coli* isolated from the environment, *Acinetobacter baumannii* AB1, and *Klebsiella pneumoniae* K3 could also be easily lysed. It was also capable of lysing several strains of pathogenic *Salmonella* isolated from the environment, even on Gram-positive bacteria *Staphylococcus aureus*, indicating that XFII is a broad-spectrum endolysin.

**Table 1:** The lytic spectrum of endolysin XFII

Species name	Strain name	Lytic activity^a^
*E. coli*	JM109	+ + + +
*E. coli*	DH5α	+ + +
*E. coli*	BL21 (DE3)	+ +
*E. coli*	MinO157	+ +
*E. coli*	19019	+ + + +
*E. coli*	19023	+ + + +
*E. coli*	19017	+ + +
*E. coli*	19082	+ + +
*E. coli*	19088	+ +
*E. coli*	19065	+
*E. coli*	19092	+
*E. coli*	19075	+
*E. coli*	19,015	+
*E. coli*	19048	–
*E. coli*	19083	–
*E. coli*	19087	–
*Salmonella* sp*.*	XFII-1	+ +
*Salmonella* sp.	XZ-5	+ +
*Salmonella* sp*.*	LY-2G	+ +
*Salmonella* sp.	XG-2	+ +
*Salmonella* sp.	18030	+
*Salmonella* sp.	18031	+ +
*Acinetobacter baumannii*	AB1	+ + +
*Klebsiella pneumoniae*	K3	+ + +
*Pseudomonas aeruginosa*	PA01	+ +
*Staphylococcus aureus*	ATCC 25923	+

^a^Relative lytic activity from 10 to 30%, “ + ”; lytic activity from 31 to 50%, “ +  + ”; lytic activity from 51 to 70%, “ +  +  + ”; lytic activity from 71 to 100%, “ +  +  +  + ”; and lytic activity lower than 10%, “–”

### Bactericidal activity of endolysin XFII under eutrophic conditions

**Fig. 7:**
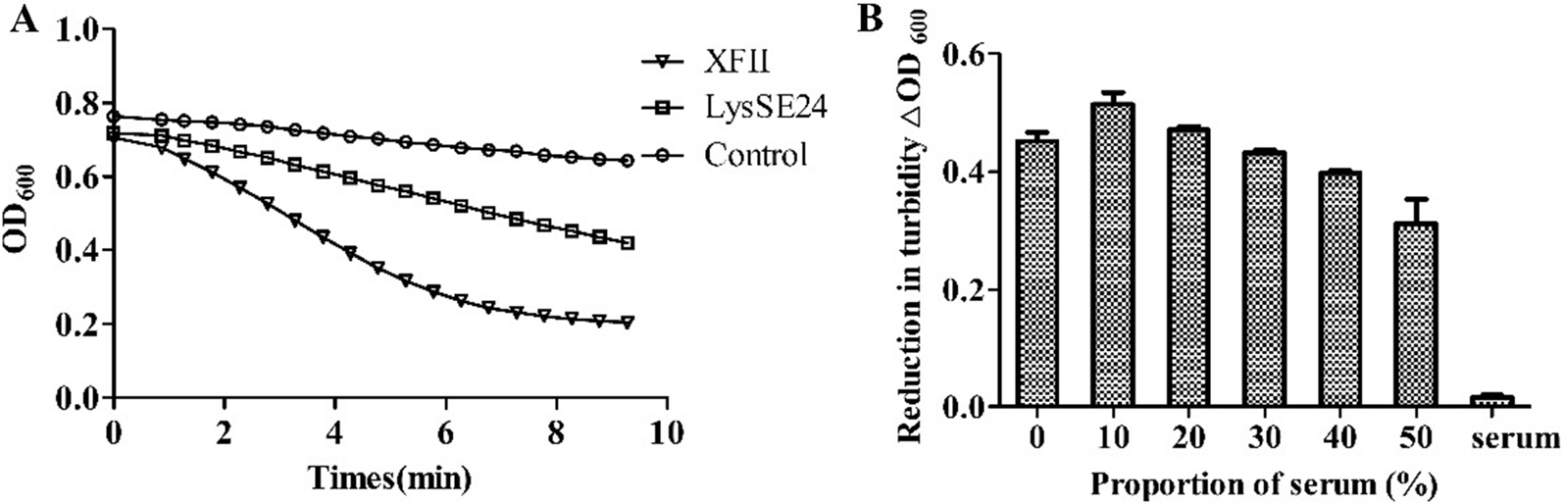
Effects of various eutrophication factors on the lytic activity of recombinant XFII. **A** The bactericidal activities of endolysin XFII and LysSE24 in LB medium. **B** The effect of rabbit serum on endolysin XFII

Although numerous endolysins have strong bactericidal activity in simple buffer solutions, the effectiveness of endolysins tend to be reduced under bacterial growth- conditions, which limits their application. Therefore, the bactericidal activity of endolysin XFII was tested in complex media including LB medium and serum medium. The results are shown in Fig. 7A. The activity of endolysin XFII against *E. coli* JM109 was slightly inhibited in LB medium. However, LysSE24, a recently reported *Salmonella* endolysin with a sequence very similar to XFII, was severely inhibited in LB medium and has almost no bactericidal activity (Fig. 7A). To test the effect of rabbit serum on endolysin XFII, the addition of rabbit serum at different proportions was added to the reaction system. The results showed that the enzyme activity was increased by 13.8% in 10% serum system, while the enzyme activity was inhibited by 31.2% when 50% serum was added (Fig. 7B).

### Synergistic bactericidal effect of chitosan and XFII

**Fig. 8:**
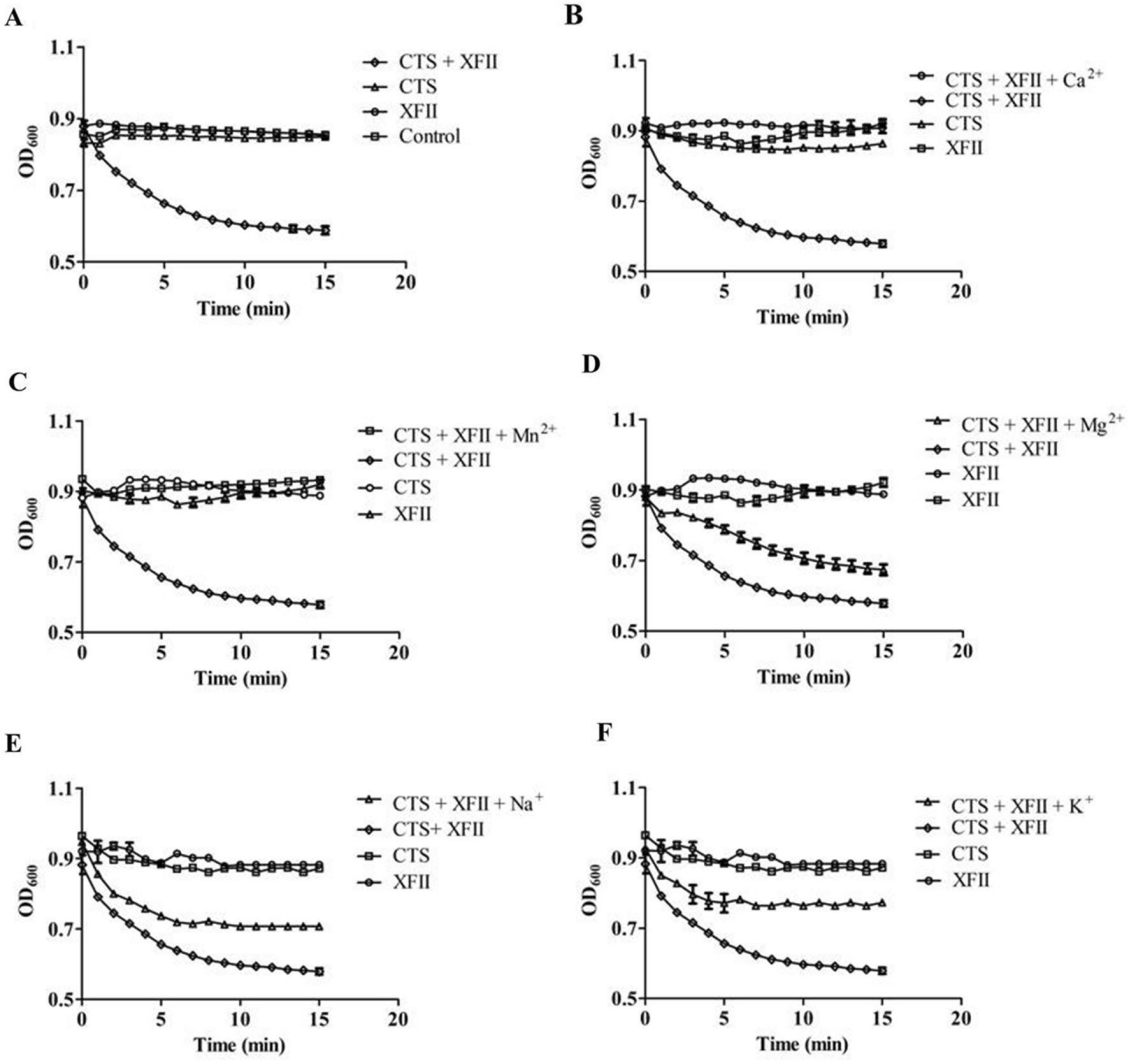
Lytic activity of XFII and chitosan on *E. coli* JM109. Enzymatic lytic activity of formulation formed by chitosan and endolysin against *E. coli* JM109 without EDTA pretreatment (A). The lytic activity of the combined formulation of XFII and chitosan against *E.coli* JM109 was determined in the presence ions of Ca^2+^ (B), Mn^2+^ (C), Mg^2+^ (D), Na^+^ (E), and K^+^ (F). CTS, Chitosan; XFII, Endolysin XFII

The synergistic effect of endolysin XFII and different antimicrobial agents on *E. coli* JM109 without EDTA pretreatment was tested to address the problem of outer membrane barriers that make it difficult for endolysins to function. Surprisingly, there was synergistic bactericidal effect between chitosan and XFII, while there was no synergistic effect of XFII and other antimicrobial agents, including nisin, tea polyphenols, ε-polylysine, potassium sorbate, sodium diacetate, hypochlorous acid, and sodium hypochlorite (data not shown). The concentration and proportion of chitosan with endolysin were optimized, the result showed that 60 μg/mL endolysin with 0.375 mg/mL chitosan had the optimal synergistic effect, and the OD_600_ decreased from 0.88 to 0.58 within 10 min (Fig. 8A). Neither endolysin XFII alone, nor Tris–HCl buffer, nor chitosan had a lytic effect, with the turbidity curve of *E. coli* JM109 showing a horizontal trend.

Since positive ions can affect the stability of the outer membrane and shield the negative charge from the surface of the cell wall, different positive ions were added to the reaction system to detect their effect on synergistic lytic activity of XFII and chitosan on *E. coli* JM109 without EDTA pretreatment. The results showed that the addition of the cations Na^+^, Mg^2+^, and K^+^ had a negative effect on the synergistic bactericidal activity, which was approximately 50% of its original level (Fig. 8D–F). It is worth noting that the addition of Ca^2+^ and Mn^2+^ resulted in a complete loss of the synergistic bactericidal activity in the case of *E. coli* JM109 without EDTA pretreatment (Fig. 8B, C).

## Discussion

In this study, a gene encoding an endolysin from the *Salmonella* phage XFII-1, named XFII, was identified and successfully over-expressed in *E. coli* BL21. The expression of endolysin was more than 100 mg/mL by optimizing the induction conditions (Fig. 1B). According to the literature, The yields were very uneven ranging from 0.5 to 80 mg/L [40–43]. The high yield characteristics of endolysin XFII will greatly reduce production costs in its industrial production and facilitate its subsequent application. Endolysin XFII exhibited rapid and strong lytic activity against *E. coli* JM109 after EDTA pretreatment, and a remarkable decrease in turbidity was observed for the first 5 min (Fig. 3A). In addition, we found that 0.5 μg/mL XFII could kill 5.3 log unit bacteria in 20 min when incubated with about 10^9^ exponential cells of host bacteria JM109 (Fig. 3B). Almost all the bacterial cells were removed after treatment with XFII that exceeded 0.5 μg/mL for 3 min (Fig. 4C, F). In TEM analysis, rupture or degradation of the bacterial cell membrane of *E. coli* treated by the endolysin XFII was also confirmed (Fig. 4C, F). Endolysin XFII was optimally effective at a concentration of 0.5 μg/mL. The bactericidal concentrations of *Salmonella* endolysin reported recently were further compared (Additional file 1: Table S1). At 0.5–0.7 OD reduction in bacterial turbidity, the lowest bactericidal concentration is 1.7 μg/mL of LysSE24 [34], which is much higher than the concentration of XFII (0.5 μg/mL). To our knowledge, the *Salmonella* endolysin LysSE24 has high lytic activity and is more tolerant of high temperatures than previously reported endolysins [34]. To compare the activity and thermotolerance between the endolysin XFII and LysSE24, LysSE24 was also expressed in our lab. Consistent with previous reports, the lysis effect of XFII and LysSE24 on *E. coli* at a concentration of 1.7 μg/mL was compared in Fig. S2A, the lytic activity of XFII was significantly higher than that of LysSE24. The bactericidal activity of endolysin XFII was nearly constant when treated at 70 °C for 2 h, and was about 54.8% when treated at 80 °C for 2 h (Fig. 6A). The thermal stability of LysSE24 (prepared in our laboratory) was determined using the same methods as for XFII (Additional file 1: Fig. S2B). Both XFII and LysSE24 had similar activity when treated at temperature below 50 °C for 2 h. While the activity of XFII was a bit higher than that of LysSE24 at 60–80℃. Simulation of food processing conditions prior to packaging and storage, 20–28℃ and 4℃ were chosen in the present study to represent room temperature and cold storage temperature, which could be kept for 16 and 175 days respectively (Fig. 6B, C). Moreover, Endolysin XFII was very stable over a wide pH range (7.0–11.0) ( Fig. 5B). All of these showed that XFII is very stable suggesting that it has unique advantages for preservation and transportation.

Since the practical application environment of endolysin is mostly eutrophic, and the in vivo environment also contains serum and various nutrients, the bactericidal activity of endolysins under eutrophic conditions is particularly important. However, the bactericidal activity of most endolysins was significantly reduced or their killing power was lost under eutrophic conditions [36]. Here we found that *Salmonella* endolysin XFII’s enzyme activity was increased by 13.8% in a 10% serum system compared to the activity without serum addition (Fig. 7B). Furthermore, XFII also has a certain activity in LB medium (Fig. 7A). It was the first reported that *Salmonella* endolysin has bactericidal activity under eutrophic conditions, which will have a advantage not only for environmental sterilization, but also for food disinfection and in vivo sterilization.

Notably, the peptidoglycan of many Gram-negative bacteria is identical [44]. Endolysin XFII was highly effective against many EDTA-pretreated Gram-negative bacteria, including *E. coli* DH5α, *E. coli* JM109, *Acinetobacter baumannii*, *Klebsiella pneumoniae* K3, and several pathogenic *E. coli and Salmonella* isolated from the environment (Table 1). However, the endolysin of Gram-negative bacteria often requried a combination of endolysins with outer membrane permeabilizer [45, 46]. In this study, EDTA was used for pretreatment to facilitate the entry of the endolysin into the peptidoglycan. The pretreatment with the cytotoxic outer membrane permeabilizer EDTA not only has a disruptive effect on the outer membrane of pathogenic bacteria, but also has certain side effects on animals. A strategy of synergistic compounding of the endolysin with various antimicrobial agents to kill bacteria without pretreatment has been proposed. The combination of nisin (a bacteriocin currently used as a bio-preservative in food) and *Listeria* endolysin PlyP100 showed a strong synergy in lysing *L. monocytogenes* [47]. LysSTG2 can controlled *S. Typhimurium* in biofilms when combined with slightly acidic hypochlorous water [19]. In our study, the synergistic bactericidal effect of endolysin and various antimicrobial agents (nisin, tea polyphenols, ε-polylysine, potassium sorbate, sodium diacetate, hypochlorous acid, and sodium hypochlorite) on *E. coli* JM109 without EDTA pretreatment were tested. Only the combination of endolysin XFII and chitosan showed good synergistic efficacy, in which the turbidity of the cells without EDTA pretreatment decreased from 0.88 to 0.58 within 10 min (Fig. 8A). However, the exact mode of action of the synergistic effect of endolysin and chitosan is still unknown. According to previous reports, chitosan acts often as a polycationic electrolyte that binds to negative charges on the cell wall, including lipopolysaccharides of Gram-negative bacteria, and can disrupt the integrity of the cell envelope [30]. In this study, different postive ions were added to the synergistic reaction system. The results showed that the addition of Ca^2+^ and Mn^2+^ resulted in a complete loss of the bactericidal activity of endolysin XFII combined with chitosan (Fig. 8B, C). To sum up the above, results a possible explanation for the synergistic effect was proposed. Chitosan may bind to the negative charge on the surface of the bacterial cell wall, which affects the stability of cell envelope. Then this allows the endolysin XFII to reach and degrade the peptidoglycan and subsequently kills the bacteria. It has been shown before that the addition of metal ions results in a dramatic reduction in the antibacterial activity of chitosan, probably because of complex formation between chitosan and these metal ions [31, 48, 49]. Tsai and Su also reported sodium ions (100 mM Na^+^) and divalent cations (Ba^2+^, Ca^2+^, Mg^2+^) of 10–25 mM can form complexes with chitosan, thus reducing the antibacterial activity of chitosan [49]. In this study, the addition of Ca^2+^ and Mn^2+^ may shield the negative charges on the cell envelope, so that chitosan cannot interact with the cell wall. These data support the hypothesis that chitosan does interact electrostatically with the bacterial cell surface, thus changing the assumption of membrane permeability. Chitosan is a safe compound has been applied in of the food industry [30]. The combined effect of chitosan and endolysin on Gram-negative bacteria will not only reduces the minimum inhibitory concentration of chitosan, but also avoid the use of outer membrane permeabilizers when endolysins are present. This is the first report on the spontaneous decontamination of *E. coli* using endolysin and chitosan synergistically. This novel combined treatment of endolysin and chitosan may be a promising method for controlling pathogenic bacterial contamination in the food industry.

## Conclusions

Overall, the novel globular endolysin XFII was screened and successfully expressed in *E. coli* BL21. Endolysin XFII exhibits a broad lysis spectrum, a rapid and strong bactericidal activity, good stability at high temperatures and under eutrophic conditions. Combined with chitosan, XFII could spontaneously lyse Gram-negative bacteria without pretreatment. The synergistic lytic mechanism of XFII and chitosan needs to be further studied. Moreover, the protein comes from intracellular, and it would be a bonus if it could be secreted extracellular by adding a signal peptide or other means.

## Materials and methods

### Bacterial strains and growth conditions

Phage XFII-1 was obtained from the liver of broiler chickens in farms (from Shandong province in China). The strains and pathogens used to test the antimicrobial spectrum in this work were listed in Additional file 1: Table S2. Competent cells of *E. coli* DH5α, BL21 (DE3) bought from Weidi biotechnology (Shanghai, China) were grown in Luria–Bertani (LB) broth.

### Cloning, over-expression, and purification of recombinant XFII

Preparation of phage XFII-1 and observation of bacteriophage plaques used the double-layer agar plate method [46]. The phage solution was filtered through a 0.22 µm sterile filter to obtain purified phage for genome extraction. The gene encoding the endolysin was amplified using a pair of primers XFII-F (CGCAAGCTTATGTCAAACCGAAACATCAG (the underlined bases encode HindIII)) and XFII-R (TATCTCGCGCTTAGCAGCGCGCCCTACAGC (the underlined bases encode XhoI)) and cloned into the vector pET-29b( +) to obtain the expression plasmid pET29b-XFII (Additional file 1: Fig. S1). The *E. coli* BL21 (DE3) transformants were further obtained and incubated at 37 °C to an OD_600_ of 0.6–0.8. Appropriate IPTG (1 mM) was added into the culture to induce expression of XFII at 16 °C for 4 h. Cells were collected by centrifugation (6800 × *g*, 4 °C, 10 min) and suspended in 10 mL lysis buffer containing 50 mM sodium phosphate (pH7.0) and 300 mM NaCl. Then disrupted by sonication for 99 cycles of 4 s pulse and 8 s pause using an ultrasonic disruptor JY92 II (Ningbo Scientz Biotechnology Co., LTD, China). Native protein extracts (His-tag at C-terminus) in the supernatant after centrifugation at 12,000 × *g* for 20 min were obtained and then purified with HisPur Ni–NTA spin columns. The purity of the endolysin was checked by SDS-PAGE [50] and protein concentration was determined by the Bradford protein assay method [51].

### Bioinformatic analysis of endolysin XFII

The functional domain analysis of XFII endolysin was performed using the NCBI Conserved Domain Database (https://www.ncbi.nlm.nih.gov/Structure/cdd/wrpsb.cgi) and the COG database (https://www.ncbi.nlm.nih.gov/COG/). The ExPASy (https://web.expasy.org/ProtParam/) online tool was used to predict the physicochemical properties of XFII endolysin. The sequences similar to XFII were searched by the Basic Local Alignment Search Tool and compared by Clustal Omega Tool. The tertiary structure of the endolysin was simulated using the online tool in Alphafold2 and analyzed by PAMOL software (https://robetta.bakerlab.org/submit.php).

### Detection of bactericidal activity of XFII

The bactericidal activity of endolysin XFII was assessed using the turbidity method according to the method with a slight modification [18, 34]. Briefly, the substrate bacterium *E. coli* JM109 was cultured at 37 °C until OD_600_ nm of 0.6–0.8. The pellet was suspended in buffer (20 mM Tris–HCl (pH 8.0)), then 100 mM EDTA solution was added additionally and incubated at 37 °C for 5 min. After EDTA was removed by centrifugation, the cell was resuspended in the above buffer at approximately OD_600_ nm = 1.0. About 1 µL of the endolysin (0.1 mg/mL) was mixed with 199 μL of pretreated *E. coil* cell. The mixture was immediately incubated at 37 °C and the OD_600_ nm was measured within 30 min. With the lysis time as the abscissa and ΔOD_600_ nm as the ordinate, the lysis curve was obtained. Unless otherwise stated, subsequent experiments were performed using EDTA-pretreated *E. coli* JM109 and an endolysin concentration of 0.5 μg/mL.

In order to further to prove that the bacteria are indeed killed upon the addition of endolysin XFII, the bactericidal activity using measurement by counting colony forming units. The JM109 cells pretreated by EDTA were suspended to OD_600_ nm = 1.0 by buffer (20 mM Tris–HCl (pH 8.0)), 0.5 μg/mL endolysin was added to the mixture, which was placed at 37 °C for reaction. At the beginning of the experiment and 3, 5,20 min later, the number of viable bacteria (colony forming units, CFU) was assessed by counting the colonies.

### Transmission electron microscopic (TEM) analysis of *E. coli* treated with XFII

TEM was used to observe the effect of XFII on bacterial cell morphology. The *E. coli* JM109 strain was harvested in the logarithmic phase. The cells were pretreated with 100 mM EDTA, after washed and adjusted to an OD_600_ of 1.0. Appropriate purified endolysin XFII (final concentration of 0.5 μg/mL) or buffer only was added to bacterial cells. In addition, *E. coli* JM109 without EDTA pretreatment was used as a control. After 3 min of incubation at 37 °C, the XFII-treated and buffer-treated *E. coli* JM109 (pretreated with EDTA) and buffer-treated *E. coli* JM109 cells were washed with PBS three times. The pellets were fixed with 2.5% glutaraldehyde for 3 h, and gently washed twice with PBS. The samples were stained with 1% phosphotungstic acid for 1.0 s and examined using a FTI Tecnai G2 F20 transmission electron microscope.

### Characterization of endolysin XFII

#### Optimal pH and temperature for bactericidal activity of XFII

To determine the optimum reaction temperature for endolysin XFII, logarithmic growth stage *E. coli* JM109 cells were pre-treated with EDTA to disrupt the outer membrane, and the cell pellet was resuspended to approximately OD_600_ nm of 1.0. The optimal temperature of XFII was evaluated in the temperature range of 4–60 °C according to the standard assay. The optimal pH of XFII was determined in various buffers at pH values ranging from 3.0 to 12.0. At the optimum temperature (37 °C), the enzyme activity was measured as described above. With the reaction temperature and pH as the abscissa and the ΔOD_600_ nm of the endolysin-treated strain within 5 min as the ordinate, histograms of the optimum temperature and pH were obtained.

#### Effects of temperature on the bactericidal activity of XFII

To evaluate the effects of temperature on enzyme activity, XFII (1 μg) was incubated at different temperatures (4–80 °C) for 2 h. After each treatment, the residual activity was assayed according to the standard assay. The relative bactericidal activity was calculated as lytic activity of the treated enzyme/lytic activity of the untreated enzyme × 100%.

#### Stability of XFII at different storage temperatures

The suitability of long-term storage of endolysin XFII was tested in accordance with the method described by Wang et al., with minor modifications [52]. It was frozen at 4 °C for a few days and the residual bactericidal activity was determined by regularly sampling according to the standard assay, and the lysis curve of the storage date was obtained. The same method was used to test the bactericidal activity of endolysin XFII after storage under ambient conditions.

### Antimicrobial spectrum of endolysin XFII

*E. coli* DH5α, *E. coli* JM109, *E. coli* C43 (DE3), standard strains of *Acinetobacter baumannii*, *Klebsiella pneumoniae*, 13 strains of pathogenic *E. coli,* and 6 strains of *Salmonella* isolated from the environment were incubated to the log phase of growth, and treated with 100 mM EDTA, especially for Gram-positive bacteria *Staphylococcus aureus* without EDTA pretreatment. The XFII endolysin (0.5 μg/mL) was added to pretreated cells, and the OD_600_ nm was measured within 30 min. The lytic activity was calculated by using the formula: Relative lytic activity (%) = 100 × ΔOD_600_ nm of the mixture with XFII / the initial OD_600_ nm of the control.

### Effects of eutrophic conditions on the activity of endolysin XFII

The influences of complex media (LB medium and rabbit serum) on bactericidal activity were also determined. EDTA-pretreated logarithmic growth phase bacteria were resuspended in LB medium, and then the bactericidal activity was determined after adding endolysin. To test the tolerance of endolysin to serum, different proportions (0, 10, 20, 30, 40, and 50%) of rabbit serum were added to 100 μL of the endolysin reaction system and the bactericidal activity was assayed according to the standard assay. As controls, a cell mixture with 50% of rabbit serum that was not treated with endolysin was included in the assays. The relative lysis activity was calculated according to the optical density difference at 600 nm.

### Combination of endolysin XFII and bacteriostatic agents

The highest experimental concentrations of various antimicrobial agents (12 mg/mL chitosan, 2.0 mg/mL nisin, 1.2 mg/mL tea polyphenols, 1.0 mg/mL ε-polylysine, 4.0 mg/mL potassium sorbate, 4.0 mg/mL sodium diacetate, 40 mg/L hypochlorous acid, and 40 mg/L sodium hypochlorite) were prepared [24, 47, 53]. Different dilution concentrations of antimicrobial agents were mixed with fixed concentrations of endolysin (0.54 mg/mL) at 1:3 (v/v) to obtain the formulations. 30 µL of the above solution was mixed with 170 μL cell suspension without EDTA pretreatment for in vitro co-lysis. Tris–HCl solution, endolysin XFII alone, and chitosan solution were used to replace the formulation as negative controls. Following the method of Cha et al. [43] and with slight modifications, a preliminary exploration of the synergistic bactericidal mechanism of the formulation formed by chitosan and endolysin in vitro was performed. In brief, the optimal concentration of the formulation (30 μL) and different postive ions (Ca^2+^, Mg^2+^, Mn^2+^, Na^+^, and K^+^, with a final concentration of 0.37 mM) were added into 170 µL of *E. coli* JM109 cell suspension. The mixture was immediately incubated at 37 °C and the OD_600_ nm was measured within 30 min.

### Statistical analysis

The data in this study were the average values in triplicate and standard deviations were expressed as error bars. The statistical tests were carried out using SPSS22.0 for Windows (SPSS Inc., USA).

## Supplementary Information

**Additional file 1: Table S1. **Bacteriophage endolysins of *Salmonella* in the literature recently. **Table S2.** Bacterial strains used in the study. **Fig. S1.** The plasmid XFII in pET29b for expression of endolysin XFII in *E.coil* BL21. **Fig. S2.** Comparison of bactericidal activity **A** and thermal stability **B** between endolysin XFII and LysSE24.

## Data Availability

All data generated or analyzed during this study are included in this published article [and the additional files]. Additional information or material, such as plasmids and strains or others, are available upon request.

## Abbreviations

EDTA: Ethylene diamine tetraacetic acid
ORF: Open reading frame
*E. coli*: *Escherichia coli*
SDS-PAGE: Sodium dodecyl sulfate polyacrylamide gel electrophoresis
OD: Optical Density
TEM: Transmission electron microscope
IPTG: Isopropyl-β-d-thiogalactoside
OMP: Outer membrane permeabilizer
LB: Luria–Bertani
