# Supplementary material for: Characterization of Salmonella endolysin XFII produced by recombinant Escherichia coli and its application combined with chitosan in lysing Gram-negative bacteria
Source: Microb Cell Fact. 2022 Aug 23;21:171. doi: 10.1186/s12934-022-01894-2 (PMC9396760; doi:10.1186/s12934-022-01894-2)
Supplement: Supplementary file 1 — Additional file 1: Table S1. Bacteriophage endolysins of Salmonella in the literature recently. Table S2. Bacterial strains used in the study. Fig. S1. The plasmid XFII in pET29b for expression of endolysin XFII in E.coil BL21. Fig. S2. Comparison of bactericidal activity A and thermal stability B between endolysin XFII and LysSE24. [file 12934_2022_1894_MOESM1_ESM.docx]

**Characterization of *Salmonella* endolysin XFII produced by recombinant *Escherichia coli* and its application combined with chitosan in lysing Gram-negative bacteria**

**Authors:** Shuhang Zhang^1^, Yan Chang^1^, Qing Zhang^2^, Yingbo Yuan^1^, Qingsheng Qi^1^, Xuemei Lu^1,^ *

^1^State Key Laboratory of Microbial Technology, Shandong University, Qingdao, 266237, China

^2^Institute of Animal Science and Veterinary Medicine, Shandong Academy of Agricultural Sciences, Jinan, 250100, China

***Correspondence**: E-mail: [luxuemei@sdu.edu.cn](mailto:luxuemei@sdu.edu.cn) Tel: +0086-532-58631558

Fax: +0086-532-58631558

Table S1.Bacteriophage endolysins of *Salmonella* in the literature recently.

| **Endolysin**  **name** | **Original Phage** | **Expression vector** | **Outer membrane**  **permeabilizers** | **Effective**  **Concentration** | **Resistance temperature** | **Bactericidal activity**  **under eutrophic conditions** | **Reference** |
| --- | --- | --- | --- | --- | --- | --- | --- |
| LysSS | *S. enterica* serovar  Enteritidis phage SS3e | pET21a | Unnecessary (highly  positively charged  amino acids) | 63-250 μg/mL | / | None | [1] |
| LysPA26 | *Pseudomonas aeruginos*a | pET28b | Unnecessary (natural lysins) | 0.5 mg/mL | 50℃ | None | [2] |
| BSP16Lys | *Salmonella* phage | pET28a | 100 mM EDTA | 2.84 μg/mL | 55℃ | None | [3] |
| LysSE24 | *Salmonella* phage LPSE1 | pET-28b | 0.5% chloroform | 1.7 μg/mL | 80℃ | None | [4] |
| M4Lys | *S. enterica* serovar  Typhimurium phage BSPM4 | pETDuet-1 | / | 1 mM | / | None | [5] |
| LysSP1 | *S. Typhimurium* phage  SLMP1 | pET28a | 5 mM EDTA | 50 µg/mL | 45℃ | None | [6] |
| LysSTG2 | *Salmonella* phage STG2 | pET29b | 40 mg/L slightly  acidic hypochlorous  water | 100 µg/mL | 50℃ | None | [7] |
| LyS15S6 | *Salmonella*-virus-FelixO1 phage BPS15S6 | pET-28a | Edible  ε-poly-L-lysine (EPL) | 34.2 µg/mL | 50℃ | None | [8] |
| LysT144 | *Salmonella spp.* serovars bacteriophage LPST144 | pET28b | saturated CHCl_3_ | 2 μg/mL | 50 °C | None | [9] |
| LysWL59 | *Salmonella Typhimurium* bacteriophage LPST10 | pET-28b | 5% chloroform | 4.5 μg/mL | 80℃ | None | [10] |
| LysWL60 | *Salmonella Typhimurium* bacteriophage LPST10 | pET-28b | 5% chloroform | 348 μg/mL | 40℃ | None | [10] |

Table S2. Bacterial strains used in the study

| **Name** | **Description** | **Source** |
| --- | --- | --- |
| JM109 | *Escherichia coli*, engineered strain | Laboratory stock |
| DH5α | *Escherichia coli*, engineered strain | Laboratory stock |
| 19015 | *Escherichia coli*, environmental separation | Shandong Academy of Agricultural Sciences |
| 19065 | *Escherichia coli*, environmental separation | Shandong Academy of Agricultural Sciences |
| 19083 | *Escherichia coli*, environmental separation | Shandong Academy of Agricultural Sciences |
| 19019 | *Escherichia coli*, environmental separation | Shandong Academy of Agricultural Sciences |
| 19023 | *Escherichia coli*, environmental separation | Shandong Academy of Agricultural Sciences |
| 19017 | *Escherichia coli*, environmental separation | Shandong Academy of Agricultural Sciences |
| 19048 | *Escherichia coli*, environmental separation | Shandong Academy of Agricultural Sciences |
| 19092 | *Escherichia coli*, environmental separation | Shandong Academy of Agricultural Sciences |
| 19087 | *Escherichia coli*, environmental separation | Shandong Academy of Agricultural Sciences |
| 19082 | *Escherichia coli*, environmental separation | Shandong Academy of Agricultural Sciences |
| 19088 | *Escherichia coli*, environmental separation | Shandong Academy of Agricultural Sciences |
| 19075 | *Escherichia coli*, environmental separation | Shandong Academy of Agricultural Sciences |
| Min O157 | *Escherichia coli* O157:H7, standard strain | Shanghai Jiao Tong University |
| AB1 | *Acinetobacter baumannii*, standard strain | Laboratory stock |
| PA01 | *Pseudomonas aeruginosa*, standard strain | Laboratory stock |
| K3 | *Pneumonic klebber*, standard strain | Laboratory stock |
| ATCC 25923 | *Staphylococcus aureus,* standard strain | Laboratory stock |
| XFII-2 | *Salmonella*, environmental separation | Shandong Academy of Agricultural Sciences |
| 18030 | *Salmonella*, environmental separation | Shandong Academy of Agricultural Sciences |
| LY-2G | *Salmonella*, environmental separation | Shandong Academy of Agricultural Sciences |
| 18031 | *Salmonella*, environmental separation | Shandong Academy of Agricultural Sciences |
| XZ-5 | *Salmonella*, environmental separation | Shandong Academy of Agricultural Sciences |
| XFII-1 | *Salmonella*, environmental separation | Shandong Academy of Agricultural Sciences |


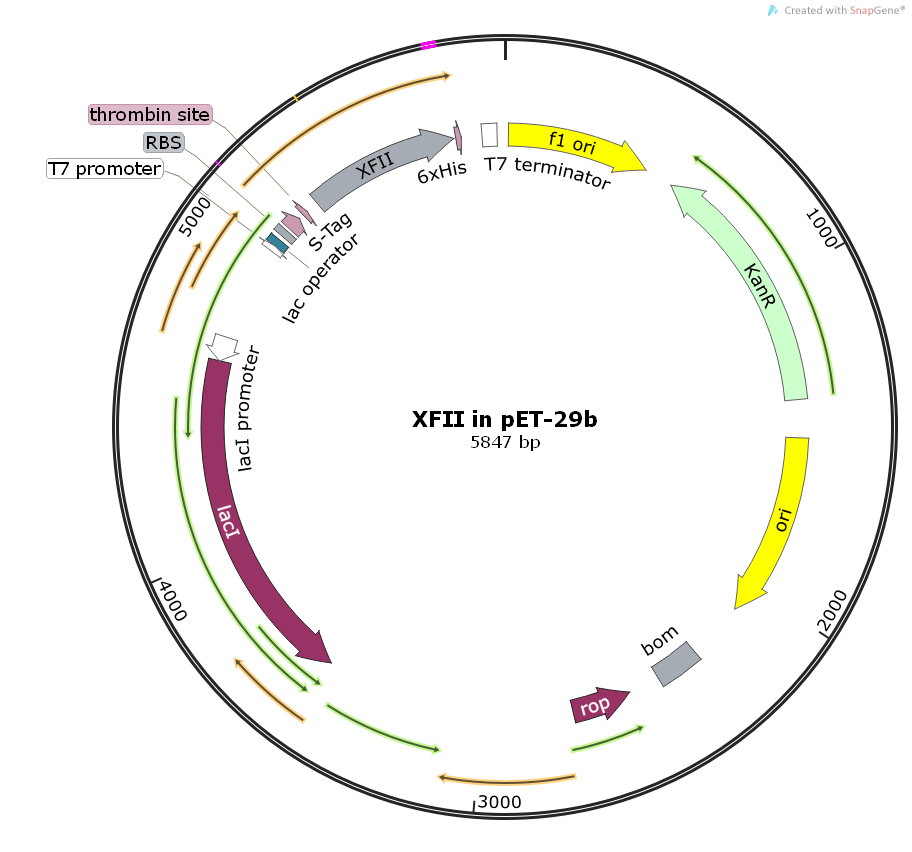


Fig. S1 The plasmid XFII in pET29b for expression of endolysin XFII in *E.coil* BL21.


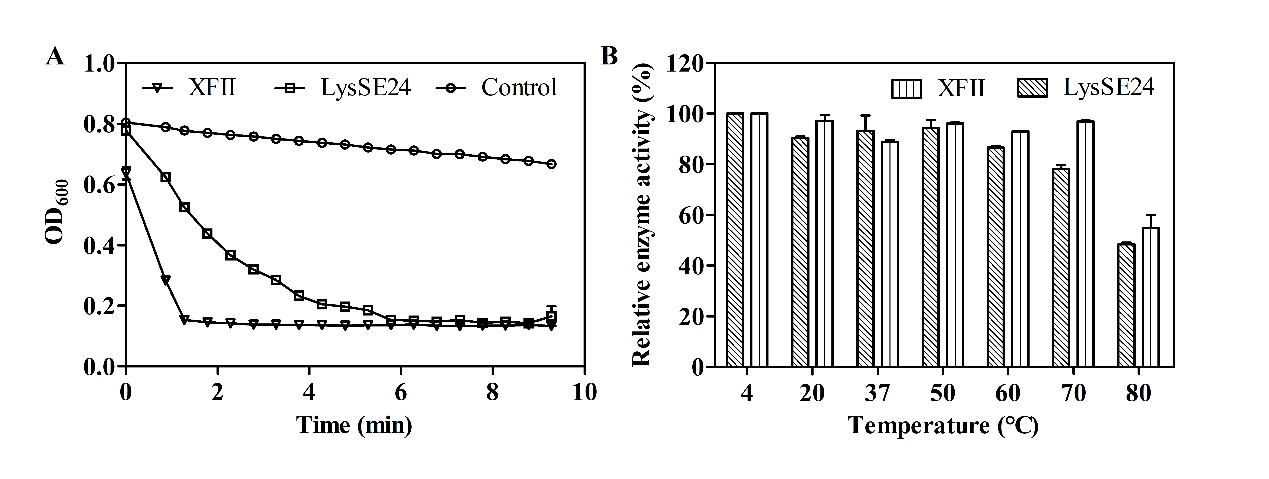


Fig. S2 Comparison of bactericidal activity (A) and thermal stability (B) between endolysin XFII and LysSE24.

**References:**

1. Kim S, Lee D, Jin J, Kim J. Antimicrobial activity of LysSS, a novel phage endolysin, against *Acinetobacter baumannii* and *Pseudomonas aeruginosa*. J Glob Antimicrob Re. 2020; 22: 32-9.

2. Guo M , Feng C , Ren J , et al. A novel antimicrobial endolysin, LysPA26, against *Pseudomonas aeruginos*a. Frontiers in Microbiology. 2017; 8:293.

3. Bai J, Yang E, Chang P, Ryu S. Preparation and characterization of endolysin-containing liposomes and evaluation of their antimicrobial activities against Gram-negative bacteria. Enzyme Microb Tech. 2019; 128: 40-8.

4. Ding Y, Zhang Y, Huang C, Wang J, Wang X. An endolysin LysSE24 by bacteriophage LPSE1 confers specific bactericidal activity against Multidrug-Resistant *Salmonella* strains. Microorganisms. 2020; 8: 737.

5. Bai J, Lee S, Ryu S. Identification and in vitro characterization of a novel phage endolysin that targets Gram-negative bacteria. Microorganisms. 2020; 8: 447.

6. Jiang Y, Xu D, Wang L, Qu M, Li F, Tan Z, et al. Characterization of a broad-spectrum endolysin LysSP1 encoded by a *Salmonella* bacteriophage. Appl Microbiol Biot. 2021; 105: 5461-70.

7. Zhang Y, Huang H, Duc HM, Masuda Y, Honjoh K, Miyamoto T. Endolysin LysSTG2: Characterization and application to control *Salmonella Typhimurium* biofilm alone and in combination with slightly acidic hypochlorous water. Food Microbiol. 2021; 98: 103791.

8. Han H, Li X, Zhang T, Wang X, Zou J, Zhang C, et al. Bioinformatic analyses of a potential *Salmonella-virus-FelixO1* biocontrol phage BPS15S6 and the characterisation and anti-Enterobacteriaceae-pathogen activity of its endolysin LyS15S6. Antonie van Leeuwenhoek. 2019; 112: 1577-92.

9. Yang Q, Ding Y, Nie R, Yao L, Wang X, Zhou M, et al. Characterization of a novel T7-like *Salmonella Typhimurium* (ATCC13311) bacteriophage LPST144 and its endolysin. LWT. 2020; 123: 109034.

10. Liu A, Wang Y, Cai X, Jiang S, Cai X, Shen L, et al. Characterization of endolysins from bacteriophage LPST10 and evaluation of their potential for controlling *Salmonella Typhimurium* on lettuce. LWT. 2019; 114: 108372.
